# Variable Resistance—An Efficient Method to Generate Muscle Potentiation: A Systematic Review and Meta-Analysis

**DOI:** 10.3390/ijerph20054316

**Published:** 2023-02-28

**Authors:** Álvaro Huerta Ojeda, Claudio Cifuentes Zapata, Guillermo Barahona-Fuentes, María-Mercedes Yeomans-Cabrera, Luis Javier Chirosa-Ríos

**Affiliations:** 1 Núcleo de Investigación en Salud, Actividad Física y Deporte ISAFYD, Universidad de Las Américas, Viña del Mar 2531098, Chile; 2 Strength & Conditioning Laboratory, CTS-642 Research Group, Department of Physical Education and Sports, Faculty of Sports Sciences, University of Granada, 18071 Granada, Spain; 3 Facultad de Salud y Ciencias Sociales, Universidad de Las Américas, Viña del Mar 2531098, Chile

**Keywords:** variable resistance, post-activation performance enhancement, strength, power

## Abstract

Variable resistance (VR) is a methodology that has shown good results in developing muscular strength and power. However, no updated information relates to the use of VR as an activation to trigger post-activation performance enhancement (PAPE). The primary objective of this systematic review and meta-analysis was to review and qualitatively describe studies published between 2012 and 2022 that used VR to generate PAPE in muscle power-dominant sports. The secondary objective was to calculate the effect size of the different power outcomes reported in the selected studies. The search was designed following the PRISMA^®^ guidelines for systematic reviews and meta-analyses and performed in the Web of Science (WOS), Scopus, SPORTDiscus, PubMed, and MEDLINE between 2012 and 2022. The methodological quality and risk of bias were evaluated with the Cochrane Collaboration tool. The main variables were the throwing speed, time in sprint tests, and jump height. The analysis was conducted with a pooled standardized mean difference (SMD) through a Hedges’ g test (95% CI). Twenty-two studies were included in the systematic review and ten in the meta-analysis, revealing a trivial effect for throwing speed (SMD = 0.06; CI = 95%: −0.23–0.35; *p* = 0.69), a small effect for the time in sprint tests (SMD = −0.37; CI = 95%: −0.72–−0.02; *p* = 0.04), and a moderate effect for jump height (SMD = 0.55; CI = 95%: 0.29–0.81; *p* ˂ 0.0001). All forms of VR used for neuromuscular activation effectively triggered PAPE. Specifically, the results showed that activation with VR generates performance increases in time, in sprint tests and jump height, and a trivial effect in throwing tests (speed and distance).

## 1. Introduction

A relevant factor for increasing physical performance is the development of muscular strength and power [1]. In this sense, it has been observed that a higher level of muscular strength is strongly associated with better strength-time characteristics. This relationship contributes directly to an athlete’s overall performance [2]. In recent years, there has been an increase in innovative methodologies that seek to optimize training time by improving muscle strength and power, avoiding overtraining, and the appearance of injuries [3,4]. Some examples of these innovations developed in recent years to increase muscle strength and power are complex training [5] and contrast training [6]. Specifically, complex training integrates resistance training with high-speed plyometric training in a single session [5,7], whereas contrast training is characterized by incorporating intense sets followed by explosive sets [6]. Regardless of the method used, when planning training loads, coaches and athletes must consider the training objectives and the magnitude of the effect of the different methodologies available [8]. These processes could consider using any available training methods to develop muscular strength and power, such as variable resistance (VR).

VR is another methodology that has shown good results for developing muscle strength and power [5,9,10]. The main characteristic of VR is the variation of intensity within the training load [11]. Based on the existing literature, loads with VR are classified in three ways: (a) intra-session variable resistance (I-SVR), a modality characterized by the modification of loads between the different series in a training session; (b) intra-series variable resistance (I-sVR), which is characterized by a load variation within the same series—part of the series is performed with one load, and the other part of the series is performed with another load; and (c) intra-repetition variable resistance (I-RVR), which is characterized by changing resistance during the repetition—in these cases, it is common to use elastic bands, chains, or functional electromechanical devices that allow varying the load in each repetition [10,12,13]. From a physiological perspective, VR increases motor neuron excitability [14], causing (i) an increased phosphorylation of myosin light chains (MLC), (ii) an increased adenosine triphosphate activity (ATP), (iii) an increased contractile capacity of the muscle fiber, and (iv) an increased motor recruitment, mainly of type II muscle fibers [15,16].

Among other purposes, VR has been used as a methodology for increasing athletic performance through the development of muscle power, specifically the generation of post-activation performance enhancement (PAPE) [17,18]. Indeed, PAPE corresponds to an enhancement of maximal voluntary (dynamic or isometric) strength, power, or speed following a conditioning contraction [18]. Based on the sequence to obtain PAPE, the literature states three phases: the first phase corresponds to the evaluation of a non-enhanced physical capacity or motor gesture; the second phase corresponds to the application of a stimulus that triggers enhancement (in this phase, a stimulus with VR could be applied); while the third phase corresponds to the re-evaluation of the physical capacity or motor gesture measured in phase 1 [17,18]. In practical terms, the level of muscle power in the third phase of the PAPE cycle is expected to increase relative to the first phase [10]. This acute increase in muscle power (PAPE) is achieved by an increase in MLC phosphorylation, an increase in the recruitment of higher-order motor units, and, at the enthesis level, a change in the angle of pennation [15,16]. At this point, it is crucial to describe the difference between “post-activation potentiation” (PAP), which corresponds to the increase in muscle force/torque production during an electrically triggered contraction, and “PAPE,” which corresponds to the improvement in peak force, power, and speed after conditioning contractions [17,18]. The precision of these concepts is recent; therefore, it is easy to find the scientific literature erroneously using the term PAP to refer to the increase of force, power, and speed in field tests [5]. Consequently, during this research’s development, PAP’s concepts may appear in textual form. However, reference is being made to PAPE.

In the research above, we described a positive relationship between using VR for neuromuscular activation and the development of PAPE [10]. Likewise, a more recent study reported the effectiveness of different activation methodologies in increasing height in vertical jumps, showing an increase in performance through PAPE [19]. From these previous studies to date [10,19], and considering the need to experiment with new methodologies for the development of muscle power [20], there is a high probability that recent research has been published reporting the use of VR as activation to trigger PAPE. Therefore, it is necessary to update and specify the number of new studies and the magnitude of each intervention’s effect (PAPE) with VR [21]. Consequently, the primary objective of this systematic review and meta-analysis was to review and qualitatively describe studies published between 2012 and 2022 that used VR to generate PAPE in muscle power-dominant sports. The secondary objective was to calculate the effect size of the different power outcomes stated in the selected investigations.

## 2. Materials and Methods

This systematic review and meta-analysis were conducted following the systematic review and meta-analysis statutes [22] and Cochrane Collaboration guidelines for assessing the risk of bias in studies. The protocol for this review is in Prospero CRD42022292026.

### 2.1. Eligibility Criteria

The literature search followed the guidelines for systematic reviews and meta-analyses [22]. For this purpose, the population (i), intervention (ii), comparators (iii), outcomes (iv), and study design (v) (PICOS) were established as follows: (i) participants included were adolescents aged 15–18 years and adults aged > 18 years with no pre-existing diseases (studies with participants with pathologies or undergoing neuromuscular rehabilitation were excluded); (ii) studies that, within their intervention protocol, had used strength training with VR methods (I-SVR, I-sVR, or I-RVR); (iii) comparators were control groups or a no-protocol intervention with VR; (iv) the outcomes were positive or negative effects on physical performance indicators (PAPE); and (v) the study design was limited to experimental studies. Studies that did not meet the eligibility criteria were excluded. Discrepancies were resolved by consensus of the investigators.

### 2.2. Sources of Information and Research

The articles selected for the systematic review and meta-analysis were published in peer-reviewed journals. The writing languages were English, Spanish, French, Portuguese, and German. The search limits were from January 2012 to November 2022. These restrictions were intended to provide evidence of a current overview of the studies analyzed. The search identified articles published in the following databases: the Web of Science (WoS), Scopus, SPORTDiscus, PubMed, and Medline. In each database, a search was performed in the title, abstract, and keyword fields. For this purpose, the following keywords were used in combination with Boolean operators AND/OR: ([“post-activation potentiation” OR “muscle potentiation” OR “muscle activation”] AND [“complex training” OR “contrast training” OR “strength training” OR “resistance training” OR “variable resistance”]). Two authors oversaw conducting the search and reviewing the studies, deciding whether the inclusion of the studies was appropriate. In case of disagreement, a third author was consulted. To see the results by keyword in each database, go to the following link: https://figshare.com/articles/dataset/Sources_of_information_and_research/22140602 (accessed on 23 February 2023).

### 2.3. Data Extraction

Data were collected using author, year, journal, objective, sample, number of participants, age, dependent and independent variable, protocol, results, performance, the experimental group (EG), and the control group (CG). One author extracted the continuous data for the meta-analysis, and another verified the correct extraction. The values were entered into a spreadsheet using Excel software and then the Review Manager software (version 5.4) (Copenhague, Dinamarca: The Nordic Cochrane Centre, The Cochrane Collaboration, 2014).

### 2.4. Risk of Publication Bias among Studies

The risk of publication bias among the studies was carried out in those parts of the systematic review and meta-analysis. Publication bias was assessed using Egger’s statistical test. This test determined the presence of bias at *p* ≤ 0.05 [23]. Funnel plots were created to interpret the overall effect, followed by an Egger statistic to confirm or refute publication bias.

### 2.5. Methodological Quality and Risk of Bias of Individual Studies

The methodological quality and risk of bias of each study selected for the systematic review and meta-analysis were assessed using the Cochrane Collaboration guidelines [24]. The verification guide was divided into six different domains: selection bias (random sequence generation, allocation concealment), performance bias (blinding of participants and staff), detection bias (blinding of outcome assessment), attrition bias (incomplete outcome data), reporting bias (selective reporting), and other types of bias (declaration of conflicts of interest). For each item, the response to a question was considered; when the question was answered with “Yes”, the bias was low; when it was “No”, the bias was high; when it was “Unclear”, a possible bias was related to lack of information or uncertainty.

### 2.6. Summary Measures and Synthesis of Results in the Studies

For the analysis and interpretation of the results in this systematic review and meta-analysis, the effect of VR training on potentiation levels in physical performance variables was examined as the primary outcome. The meta-analysis was only conducted if the selected study met the following criteria: (a) intervention with VR for developing muscle strength or power, (b) contained a CG and an EG or a second EG that allowed data comparison, and (c) post-intervention assessments. In addition, if the study design allowed more than one comparison, it was denoted with the letters a, b, c, d, e, and f, respectively. Studies that did not meet some of these criteria were only considered for the systematic review. To assess the quality of the experiments and interpret the risk of bias values, Review Manager version 5.4 was used (Copenhague, Dinamarca: The Nordic Cochrane Centre, The Cochrane Collaboration, 2014). The same software was used for the meta-analysis’s descriptive and statistical analysis. To compare the effects of VR interventions on strength development, the number of participants, standardized mean difference (SMD), and standard error of SMD were analyzed for each study. While calculating the SMD for each study, the Hedges’ g-test was used [25]. The overall effect and 95% confidence interval (CI) were calculated by weighting the SMD by inverse variance. In addition, the SMD of the EG and CG groups were subtracted to obtain the effect size (ES), which was used together with the pooled SD of change to calculate the variance (ES = [EG mean–CG mean]/SD). Cohen’s criteria to interpret the magnitude of the ES were <0.2, trivial; 0.2–0.5, small; 0.5–0.8, moderate; and >0.8, large [26].

Due to the actual heterogeneity rather than chance, the I^2^ statistic was calculated as an indicator of the total observed variation of the studies. I^2^ values are included from 0 to 100%, representing (i) a small amount of inconsistency (between 25% and 50%), (ii) a medium amount of heterogeneity (between 50% and 75%), and (iii) high heterogeneity (when the I^2^ value was greater than 75%). Specifically, low, moderate, and high adjectives would be acceptable, referring to I^2^ values of 25%, 50%, and 75%, respectively, although a restrictive categorization would not be appropriate in all circumstances [27].

## 3. Results

### 3.1. Study Selection

**Figure 1:**
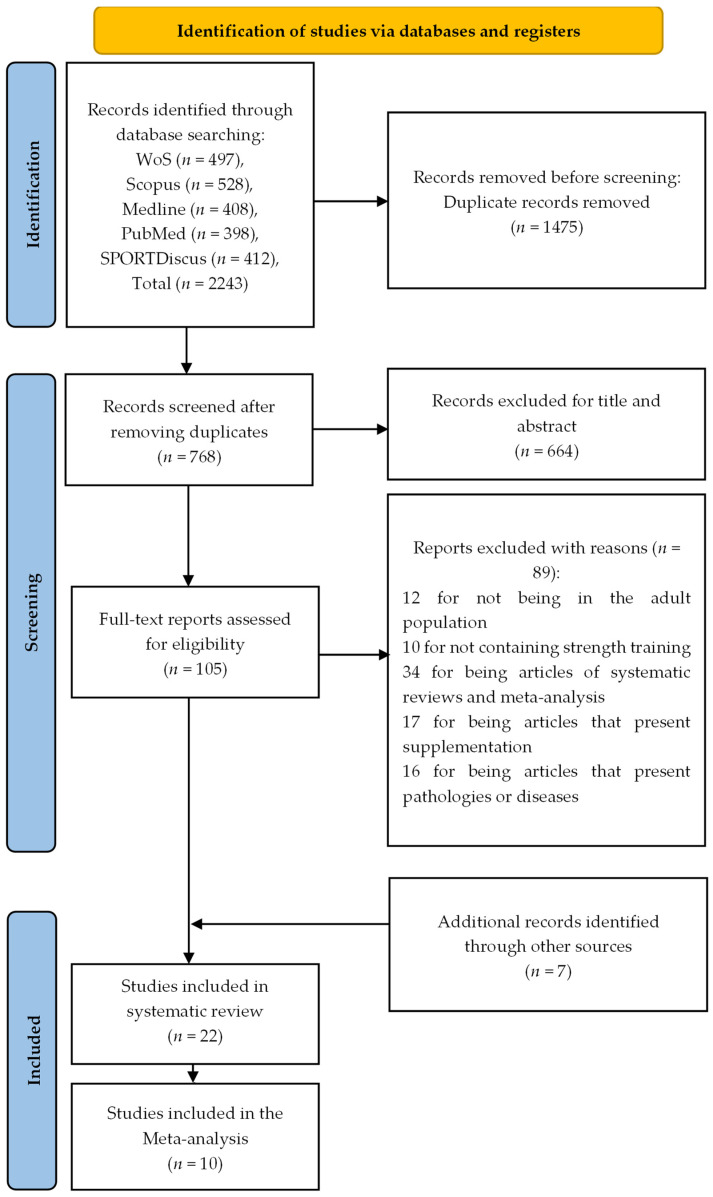
Flowchart summarizing the process of study selection.

The bibliographic search through electronic databases identified 2243 articles, of which 1475 were duplicates. The remaining 768 articles were filtered by title and abstract, leaving 104 studies to be read and analyzed in extenso. After analyzing the 104 studies, 89 were eliminated because they did not meet the inclusion criteria. Subsequently, seven articles were included by reference searching. As a result, 22 articles were included in the systematic review. Of these 22, 10 articles met the criteria for inclusion in the meta-analysis (Figure 1).

All 22 studies in the systematic review applied VR for explosive strength development. Nine studies used I-SVR [28,29,30,31,32,33,34,35,36], ten used I-sVR [37,38,39,40,41,42,43,44,45,46], and three I-RVR [47,48,49]. Likewise, 12 studies evaluated the effect of VR on jump height; of these, 10 used the countermovement jump (CMJ) [28,29,30,31,33,34,37,38,39,48] and 2 used squat jumps (SJ) [32,36] to evaluate performance. Seven studies assessed the effect of VR on sprint time; of these, five used the 30-m sprint test [31,42,43,45,46], one used the repeated-sprint ability test (RSA) [35], and one used the 9.1-m test [49]. Four evaluated the effect of VR on upper limbs PAPE [40,41,44,47]; of these, two evaluated the throwing speed [40,47] and two the throwing distance [41,44]. Finally, two studies included markers of muscle damage (creatine kinase) as an outcome [44,46]. The characteristics and effects of the different VR methods used for PAPE development are reported in Table 1.

### 3.2. Assessment of Methodological Quality and Risk of Bias of Individual Studies

**Figure 2:**
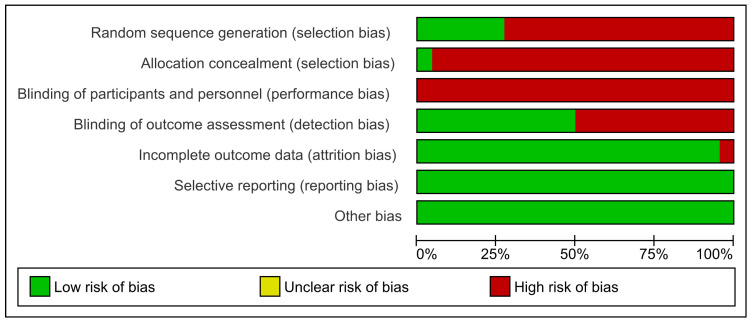
Risk of bias graph: review authors’ judgments about each risk of bias item presented as percentages across all included studies.

**Figure 3:**
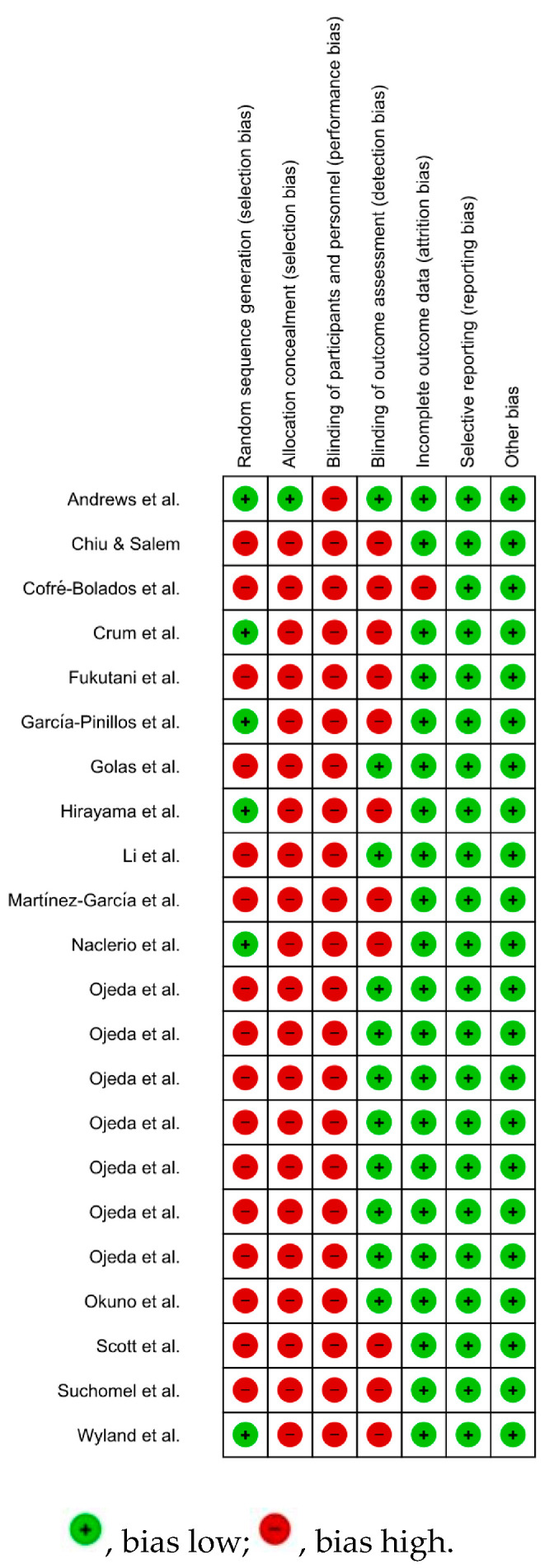
Risk of bias summary: review authors’ judgments about each risk of bias item for each included study [28,29,30,31,32,33,34,35,36,37,38,39,40,41,42,43,44,45,46,47,48,49].

The evaluation of the methodological quality and risk of bias of the 22 studies selected for the systematic review showed that the study developed by Andrews et al. [28] was the only one with a low risk of bias for the domains’ random sequence generation and allocation concealment. Likewise, all included studies showed a high risk of bias in the domain of blinding of participants and personnel [28,29,30,31,32,33,34,35,36,37,38,39,40,41,42,43,44,45,46,47,48,49]. Eleven studies showed a high risk of bias for the domain blinding of outcome assessment [29,30,31,33,36,37,38,39,47,48,49]. Only the study by Cofré-Bolados et al. [29] showed a high risk for the incomplete outcome data domain. All included studies showed a low risk of bias for the domains’ selective reporting and other biases [28,29,30,31,32,33,34,35,36,37,38,39,40,41,42,43,44,45,46,47,48,49] (Figure 2 and Figure 3).

### 3.3. Meta-Analysis

During the analysis of the selected studies, 10 met the inclusion criteria for the meta-analysis [28,29,31,34,35,39,40,46,47,49]. Consequently, these ten studies were meta-analyzed in three outcomes: throwing speed, time in sprint tests, and jump height. Two studies were considered for the meta-analysis of throwing speed [40,47]. Four studies were considered for the meta-analysis of time in sprint tests [31,35,46,49], and four were considered for jump height [28,29,34,39].

### 3.4. Publication Bias

**Figure 4:**
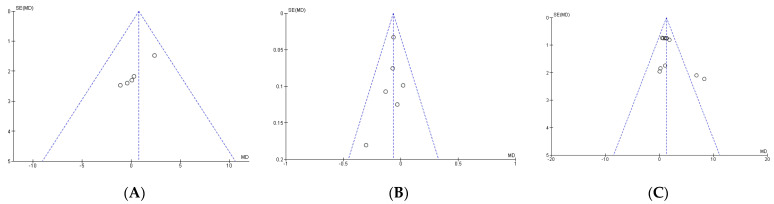
The standard error for throwing speed (**A**), time in sprint tests (**B**), and jump height (**C**). SE: standard error; SMD: standardized median difference.

Publication bias of the ten studies that were meta-analyzed was assessed using Egger’s statistical test. This test determined the presence of bias at *p* ≤ 0.05 [23]. Funnel plots were created to interpret the general effect, followed by an Egger’s statistic to confirm or refute publication bias. Egger’s analysis suggested that the primary variables did not show publication bias: A, throwing speed: z = 0.40, *p* = 0.69; B, time in sprint tests: z = 2.06, *p* ˂ 0.04; C, jump height: z = 4.14, *p* ˂ 0.0001 (Figure 4).

### 3.5. Effect of VR on Throwing Speed

**Figure 5:**
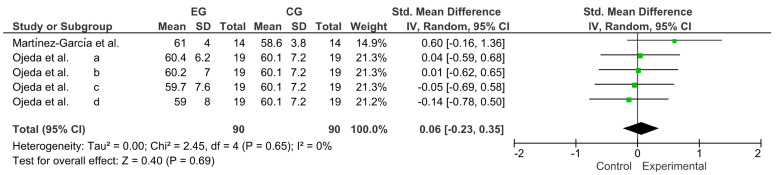
Forest plot comparing the effects of training with VR on throwing speed [40,47].

Two studies were considered for this analysis [40,47]. The research by Ojeda et al. [40] was considered as four independent studies for performing four comparisons (1a, 1b, 1c, and 1d). Consequently, to calculate the effect of VR on throwing speed, this meta-analysis considered the five comparisons as independent studies. Figure 5 shows the trivial effect of VR on throwing speed (SMD = 0.06; CI = 95%: −0.23–0.35; *p* = 0.69). The meta-analysis showed low heterogeneity among the studies reviewed (I^2^ = 0%; *p* = 0.65).

### 3.6. Effect of VR on Time in Sprint Tests

**Figure 6:**
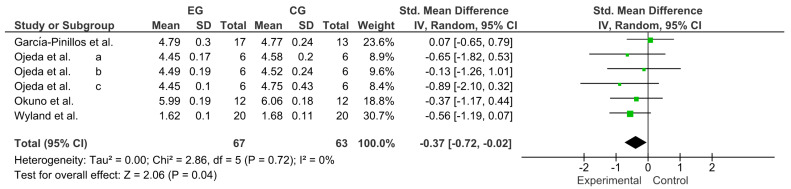
Forest plot comparing the effects of training with VR on time in sprint tests [31,35,46,49].

Four studies were considered for this analysis [31,35,46,49]. However, the study by Ojeda et al. [46] performed three comparisons (2a, 2b, and 2c). Therefore, to calculate the effect of VR on sprint time, this meta-analysis included six comparisons as independent studies. Figure 6 shows the small effect of VR on sprint time (SMD = −0.37; CI = 95%: −0.72–−0.02; *p* = 0.04). The meta-analysis showed low heterogeneity among the studies reviewed (I^2^ = 0%; *p* = 0.72)

### 3.7. Effect of VR on Jump Height

**Figure 7:**
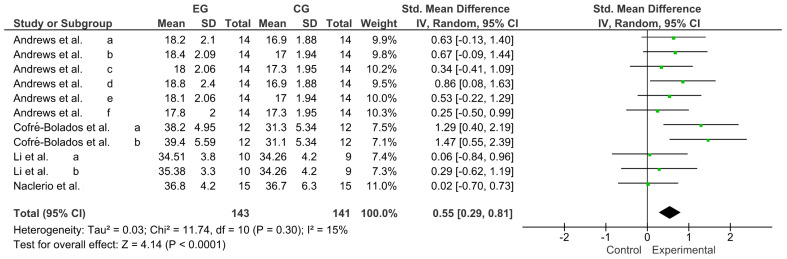
Forest plot comparing the effects of training with VR on jump height [28,29,34,39].

Four studies were considered for this analysis [28,29,34,39]. However, the study by Andrews et al. [28] performed six comparisons (a, b, c, d, e, and f), while the studies by Cofré-Bolados et al. [29] and Li et al. [34] performed two comparisons each (a and b). Therefore, to calculate the effect of VR on jump height, this meta-analysis included 11 comparisons as independent studies. Figure 7 shows the moderate effect of VR on jump height (SMD = 0.55; CI = 95%: 0.29–0.81; *p* ˂ 0.0001). The meta-analysis showed low heterogeneity among the studies reviewed (I^2^ = 15%; *p* = 0.30).

## 4. Discussion

This systematic review and meta-analysis aimed to review, qualitatively describe, and meta-analyze studies published between 2012 and 2022 that used VR to generate PAPE in muscle power-dominated sports. The systematic review and meta-analysis results show that training with I-SVR [28,29,30,31,32,33,34,35,36], I-sVR [37,38,39,40,41,42,43,44,45,46], and I-RVR [47,48,49] positively affects sprint time and jump height and has a trivial effect on throwing speed.

Scientific evidence has shown that VR generates better neuromuscular responses than other neuromuscular stimulation [5,21,50]. Indeed, it has been observed that VR stimuli cause greater phosphorylation of myosin regulatory light chains and greater excitation of the Hoffman reflex (h-reflex) than other training methodologies [16,50]. In this sense, PAPE is one of the phenomena that are increased when using VR [15,21]. Pagaduan et al. [5] analyzed the responses of the neuromuscular system after a VR stimulus, comparing them with resistance training (RT) on PAPE. At the end of the study, it was observed that training with VR generates a greater jumping capacity (PAPE) than a stimulus with RT [5]. Likewise, in a meta-analysis by Bauer et al. [50], and after different stimuli, performance was evaluated in tests with a predominance of muscular power. At the end of the study, increases in sprint performance were reported after VR stimuli compared with other strength training methods (declared as complex training by the authors). In another meta-analysis conducted by Cormier et al. [21], jumping and sprinting capacity were evaluated after stimuli with VR and other training methodologies. At the end of the study, significant differences in favor of VR were obtained (ES = 0.88 and −0.94, respectively) [21]. On the other hand, in a study by Freitas et al. [51], the effect of training with VR was also analyzed on jumping and sprinting capacity. At the end of the intervention, a medium effect was observed on sprinting (ES = 0.74) and a small effect on jumping capacity (ES = 0.45). Despite these favorable results, most research also concludes that responses to different VR stimuli, which trigger PAPE, should be observed individually and, therefore, will depend on how each athlete responds to the stimulus structure (duration, series, repetitions, pause, etc.) [52]. In this sense, it has been proven that more experienced athletes generate better responses (PAPE) to strength stimuli when compared to people less experienced in this type of training [41,52].

### 4.1. Intra-Session Variable Resistance Training (I-SVR)

Although the generation of PAPE through VR is well documented by the scientific literature [5,21,50], there are still some inconsistencies in the production of PAPE through I-SVR in high-intensity sports with a predominance of actions such as jumping and sprinting [51,53,54]. For example, Okuno et al. [35] investigated the increase in performance through the RSA test after activation through a half squat (1 × 5 × 50% 1RM + 1 × 3 × 70% 1RM + 5 × 1 × 90% 1RM), showing significant differences between EG and CG (*p* ˂ 0.01, *d* = 0.41). However, it is essential to consider that this and all results could be conditioned to the test structure (RSA), as repeated sprints could contribute to the onset of fatigue [1]. In this sense, due to the scarce scientific evidence, it is necessary to develop more research to help determine the effects of I-SVR in RSA tests [35]. On the other hand, when determining the effects of I-SVR on jumping tests, the results appear to be more consistent [28,29,34,39]. In this sense, a moderate ES has been evidenced in jumping tests after activation with I-SVR (*d* = 0.55). Likewise, I-SVR activation seems sufficient to generate PAPE in jumping tests [21,50]. Despite the existing evidence, observing individual responses to pauses, intensities, and training levels [15,16,55] is crucial when applying activation protocols with I-SVR.

### 4.2. Intra-Series Variable Resistance Training (I-sVR)

The systematic review reported ten studies that used I-sVR activation to generate PAPE [37,38,39,40,41,42,43,44,45,46]. Two observed PAPE through throwing tests [40,41], four through sprints [42,43,45,46], and three through CMJ [37,38,39]. In addition, one of the studies reported the post-exertion variations of CK after stimulus with I-sVR [44]. Based on the findings, with the description of each article, all activations with I-sVR could be replicated. For example, Ojeda et al. [45] established the neuromuscular activation on movement velocities (back squat: 1 × 5 × 22% 1RM (equivalent to 1.1 m·s^−1^) + 1 × 4 × 60% 1RM (equivalent to 0.6 m·s^−1^)) and then PAPE testing through a 30-m sprint, reporting that the first part of the series (22% 1RM) allows activation of the neural system without the presence of fatigue. In comparison, the second part of the series (60% 1RM) enables the activation of type II fibers. The researchers also reported that the athletes had to activate I-sVR at the highest possible speed to trigger PAPE [45]. This last condition would also make it possible to shorten the stimulus times per session [45,56]. However, considering that most of the studies reported results in the military population and female athletes, it is essential to note that the results of these studies are not always comparable to those of the military population [40,41,42,43,44,45,46]. There is still a need for further research relating activation with I-sVR on PAPE in different populations, both for responses—acute effects—and adaptations—chronic effects.

### 4.3. Intra-Repetition Variable Resistance Training (I-RVR)

The systematic review reported three studies with the activation of I-RVR to produce PAPE [47,48,49]. In this sense, Martínez-García et al. [47] applied an activation with I-RVR in a unilateral chest press (1 × 5—starting speed of 0.6 m s^−1^ and a final speed of 0.9 m s^−1^) on throwing speed. At the end of the study, the investigators found no significant difference in the throwing speed (*p* > 0.194, ES = 0.088). In parallel, Scott et al. [48] applied an activation with I-RVR using hex bar deadlift and back squat exercises (1 × 3 × 70% 1RM + elastic band (0–23% 1RM), rest 30 s, CMJ, rest 90 s, CMJ, and rest 180 s, CMJ). It showed positive results between the baseline and the CMJ performed 30 s after activation with I-RVR (BL vs. 30 s, *p* = 0.003). Likewise, Wyland et al. [49] applied an activation with I-RVR using back squats with 30% of the total load coming from accommodating, evidencing significant increases in 9.1-m sprint performance (9.1-m sprint: 0 min vs. 4 min post activation *p* = 0.002). Based on the background and results analyzed in this systematic review and meta-analysis, activation with I-RVR generates greater neuromuscular activation, producing increased muscle power levels [57]. However, as with other training methods, it is essential to consider individual responses to these neuromuscular activation stimuli [47].

### 4.4. Variable Resistance and Throwing Speed

Currently, there is evidence of the use of constant resistance as a methodology to generate PAPE [58,59]. These studies have shown that significant throwing speed increases by using constant resistance as neuromuscular activation [60,61,62]. However, only two studies are observed when analyzing VR as an activation to increase throwing speed (PAPE) [40,47]. In this sense, both the research of Ojeda et al. [40], which used I-sVR as activation, and the study of Martínez-García et al. [47], which used I-RVR as activation, reported non-significant changes in throwing speeds (*p* > 0.05), concluding the importance of individualized analysis for each study. In this way, observing whether athletes are responsive or non-responsive to the different VR methodologies would be possible. Similarly, Ojeda et al. [41] analyzed throwing distances after I-sVR activation, comparing professional vs. amateur athletes. At the end of the study, the researchers reported that professional athletes significantly increased throwing distance (*p* ˂ 0.05). In contrast, amateur athletes evidenced fatigue before the same activation with I-sVR, considerably decreasing the throwing distance evaluated at baseline (*p* ˂ 0.05) [41]. Although the meta-analysis showed a trivial effect of VR on throwing speed (ES = 0.06), it is also important to note that the number of studies included in this analysis was low (two studies). In addition, the methodological designs used in these studies do not allow us to conclude the actual effect of VR as an activation to generate PAPE.

### 4.5. Variable Endurance and Time Performance in Sprint Tests

Most sports involve accelerations, speed changes, direction changes, and sprints, especially team sports [11]. In this sense, other research has reported a positive relationship between increases in muscle strength and power with an increase in speed [11,54]. In this context, evidence links VR to better performance in sprint tests [35,46,49]. Research of Okuno et al. [35], which used I-SVR as activation, the investigation of Ojeda et al. [46],which used I-sVR as activation, and the research of Wyland et al. [49], which used I-RVR as activation, reported significant increases in time in sprint tests (*p* ˂ 0.05), concluding the benefit of this form of activation to trigger PAPE in different sports modalities. In this line, the meta-analysis showed a small effect of VR on time in sprint tests (ES = −0.37), evidencing an increase in performance (PAPE) after using VR as activation. This increase in sprint test performance (PAPE) after activation with VR seems to be due to the phosphorylation of MLCs. This event stimulates ATPase activity (an enzyme regulated by the thin actin filament) [16]. This enzyme, in turn, alters the structure of the myosin head away from the thick myosin filament and towards the actin filament, causing intramyocellular events that increase cross-bridge velocity and enhance muscle contraction [21,50,63].

### 4.6. Variable Resistance and Jumping Capacity

Scientific evidence shows contradictory results of conventional training for developing muscular strength and power in jumping capacity [60,62,64]. However, the use of VR as an activation to increase jumping ability (PAPE) shows encouraging results [28,29,34]. Indeed, the research of Andrews et al. [28], Cofré-Bolados et al. [29], and Li et al. [34], which used I-SVR as activation, reported significant increases in jumping ability (PAPE) following activation with I-SVR (*p* ˂ 0.05). Specifically, the meta-analysis showed a moderate effect of I-SVR on jumping capacity (ES = 0.55). It appears that, as in the other outcomes reported in this study, the increase in yield (PAPE) following VR activation is related to the phosphorylation of MLCs [16] and all the intramyocellular events described above [21,50,63].

### 4.7. VR Considerations for Generating PAPE

The different forms of VR described in this systematic review and meta-analysis showed that this training methodology has characteristics that allow adequate activation to trigger PAPE. However, specific considerations must be taken into account to achieve this goal [52]: (a) using VR in professional or experienced strength-training athletes [41,65]; (b) when training with women, considering a longer pause between the activation and the next exercise [45,46]—this will increase the chances of triggering PAPE in this group of athletes; (c) considering the advances in the quantification and control of training loads, future research needs to use reliable devices for the application of VR on PAPE [59,66]; and (d), when applying activation protocols with VR (I-SVR, I-sVR or I-RVR), individual responses should be observed, considering the pauses, intensities, and training level of each athlete [15,16,55].

### 4.8. Limitations

The limitations observed during the development of the systematic review and meta-analysis were as follows: (a) lack of randomized controlled trials (adequate statistical power, characteristics of participants, blinding of outcome measures), (b) the inclusion of studies with different languages, (c) use of different terminology to refer to variables and outcomes, including the use of PAP and PAPE in the different included studies, and (d) the scarcity of studies comparing standard PAP and/or PAPE protocols with VR protocols.

## 5. Conclusions

All forms of VR used for neuromuscular activation (I-SVR, I-sVR, and I-RVR) effectively triggered PAPE. From a physiological point of view, the increase in performance after VR activation is due to MLC phosphorylation, which increases cross-bridge velocity and enhances muscle contraction. Specifically, the results showed that activation with VR generates a small increase in sprint performance, a moderate increase in jump performance, and a trivial effect on throwing performance (speed and distance). Finally, the scarce information that relates VR and possible PAPE in throwing tests opens the possibility of studying the effect of I-SVR, I-sVR, or I-RVR using different intensities and electronic devices that allow reliable quantification of the training loads.

## 6. Future Lines of Research and Practical Applications

From a practical point of view, working with VR is a valid alternative to increase muscle power. However, some considerations must be taken into account when training with VR. (a) Based on the existing literature, to trigger PAPE, one should stimulate with loads around 80% of 1RM. These loads allow for stimulating type II fibers. (b) Currently, functional electromechanical devices allow reliably quantified training loads, especially I-RVR. In this context, using elastic bands or chains has shown good results, but monitoring training loads with these implements is difficult. (c) If functional electromechanical devices are not available to train with I-RVR, it is suggested to trigger PAPE with I-SVR or I-sVR. This type of VR linear transducer (encoder) can monitor running speeds and, thus, accurately control and/or quantify training loads.

As future lines of research, researchers and coaches are invited to test further VR protocols in power zones between 0.6 and 0.9 ms^−1^ vertical bar velocity and use functional electromechanical devices for all forms of VR in both men and women.

## Figures and Tables

**Table 1:** Characteristics of the studies that connect VR with PAPE.

Author	Objective	Participants	Variables	Test	Protocols	Outcomes	Performance
Intra-Session Variable Resistance							
Andrews et al. [28]	To investigate if a unilateral resistance training-type conditioning exercise session would elicit a non-local (crossover) facilitation of jump performance.	University athletes: M = 8 (21.2 ± 0.4 years) W = 6 (21.3 ± 1.8 years) EG1 = 14 EG2 = 14 CG = 14 The participants completed three conditions on separate days in random order	IV: I-SVR CT DV: Explosive strength of lower limbs	CMJ: h (cm)	EG1 (dominant leg): Bulgarian split squat: 1 × 5 × 50% 1RM, rest 3 min + 1 × 2 × 70% 1RM, rest 3 min + 1 × 1 × 90% 1RM, + DJ + CMJ (1-, 5-, and 10-min post-treatment). EG2 (non-dominant leg): Bulgarian split squat: 1 × 5 × 50% 1RM, rest 3 min + 1 × 2 × 70% 1RM, rest 3 min + 1 × 1 × 90% 1RM, + DJ + CMJ (1-, 5-, and 10-min post-treatment). CG: Warm-up + rest 8 min + DJ + CMJ (1-, 5-, and 10-min post-treatment).	EG1–CMJ (cm): Pair a: pre-test = 17.0 ± 1.94 vs. min 1 = 18.2 ± 2.10, *p* = 0.008 Pair b: pre-test = 17.0 ± 1.94 vs. min 5 = 18.4 ± 2.09, *p* = 0.011 Pair c: pre-test = 17.0 ± 1.94 vs. min 10 =18.0 ± 2.06, *p* = 0.013 EG2–CMJ (cm): Pair d pre-test = 18.6 ± 2.10 vs. min 1 = 18.8 ± 2.40, *p* = 0.034 Pair e: pre-test = 18.6 ± 2.10 vs. min 5 = 18.1 ± 2.06, *p* = 0.20 Pair f: pre-test = 18.6 ± 2.10 vs. min 10 = 17.8 ± 2.00, *p* = 0.05 CG–CMJ (cm): Pair g: pre-test = 17.2 ± 1.95 vs. min 1 = 16.9 ± 1.88, *p* > 0.05 Pair h: pre-test = 17.2 ± 19.5 vs. min 5 = 17.0 ± 1.94, *p* > 0.05 Pair i: pre-test = 17.2 ± 1.95 vs. min 10 = 17.3 ± 1.95, *p* > 0.05	EG1: ↑ EG2: ↓ CG: ↔
Cofré-Bolados et al. [29]	To determine the PAP, three protocols specifically warm.	Athletes: M = 12 (23.6 ± 2.1 years) EG1 = 12 EG2 = 12 EG3 = 12 The participants completed three conditions on separate days in random order	IV: I-SVR CT DV: Explosive strength of lower limbs	CMJ: h (cm)	EG1 multi jumps: 7 jumps (box of 30 cm) + 7 jumps (mini hurdles of 35–40 cm) + 5 jumps dominant leg + 5 jump non-dominant leg + 10 running strides + 5 long jump without running + 5 DJ (h 60 cm) + 1 sprint 10 m + 1 sprint 20 m + 5 pushups. EG2 loaded half squat: 1 × 10 × 20 kg with jump, rest 1 min, 1 × 4 × 40% 1RM with jump, + 1 × 4 × 70% 1RM + 1 × 3 × 80% 1RM + 1 × 3 × 60% 1RM + 1 sprint 10 m + 1 sprint 20 m + 5 pushups. EG3 half squat and contrast jump: 1 × 5 × 50% 1RM + 10 s jumps (box of 30–35 cm), rest 1 min, 1 × 4 × 30% 1RM + 4 CMJ + 1 × 4 × 50% 1RM + 4 CMJ + 1 × 3 × 85% 1RM + 6 jump (mini hurdles of 40 cm) + 1 sprint 10 m + 1 sprint 20 m + 5 pushups.	EG1: CMJ (cm): 31.3 ± 5.34 EG2: CMJ (cm): 38.2 ± 4.95 EG3: CMJ (cm): 39.4 ± 5.59	EG1: ↔ EG2: ↔ EG3: ↑
Fukutani et al. [30]	To examine the influence of the intensity of squat exercises on the subsequent jump performance and the magnitude of PAP.	Healthy Olympic lifters: M = 8 (19.8 ± 1.3 years) EG1 = 8 EG2 = 8 The participants completed two conditions on separate days in random order	IV: I-SVR CT DV: Explosive strength of lower limbs	CMJ: h (cm)	EG1 heavy condition—squat exercise: CMJ × 3 + 1 × 5 × 45% 1RM, rest 2 min + 1 × 5 × 60% 1RM, rest 2 min + 1 × 3 × 75% 1RM, rest 2 min + 1 × 3 × 90% 1RM, rest 1 min + CMJ × 3. EG2: moderate condition—squat exercise: CMJ × 3 + 1 × 5 × 45% 1RM, rest 2 min + 1 × 5 × 60% 1RM, rest 2 min + 1 × 3 × 75% 1RM, rest 1 min + CMJ × 3.	EG1: CMJ (cm): pre = 46.3 ± 7.8 vs. post = 51.1 ± 8.1, *p* = 0.012 EG2: CMJ (cm): pre = 47.9 ± 8.5 vs. post = 49.7 ± 8.2, *p* = 0.001	EG1: ↑ EG2: ↑
García-Pinillos et al. [31]	To determine the effects of a 12-week contrast training program (isometric + plyometric), with no external loads, on young soccer players’ vertical jump, kicking speed, sprinting, and agility skills.	Semiprofessional soccer players: EG: 17 (15.4 ± 1.2 years) CG: 13 (16.3 ± 1.5 years)	IV: I-SVR DV: Explosive strength of upper limbs	CMJ: h (m) peak power (W·kg^−1^) Speed test (5, 10, 20, and 30 m)	EG: A 12-week contrast training program	EG vs. CG CMJ (m): ANOVA: *p* ˂ 0.05 Post hoc: EG: pre-test (0.42 ± 0.06) vs. post-test (0.45 ± 0.04), *p* ˂ 0.001 CG: pre-test (0.45 ± 0.03) vs. post-test (0.46 ± 0.03), *p* = 0.058 EG vs. CG peak power (W·kg^−1^): ANOVA: *p* ˂ 0.05 Post hoc: EG: pre-test (29.0 ± 6.8) vs. post-test (31.5 ± 6.5), *p* = 0.044 CG: pre-test (31.5 ± 5.2) vs. post-test (32.1 ± 5.1), *p* = 0.642 EG vs. CG speed test (5 m): ANOVA: *p* > 0.05 EG vs. CG speed test (10 m): ANOVA: *p* > 0.05 EG vs. CG speed test (20 m): ANOVA: *p* ˂ 0.05 EG vs. CG speed test (30 m): ANOVA: *p* ˂ 0.05	EG ↑ CG: ↔
Golas et al. [32]	To evaluate the changes in RFD, RPD, and jump height during a complex training session consisting of the barbell half squat.	Ski jumping: M = 16 (23.0 ± 8.0 years) 60% 1RM = 16 70% 1RM = 16 80% 1RM = 16 90% 1RM = 16 100% 1RM = 16 Participants completed all the conditions in the same session	IV: I-SVR Load (60, 70, 80, 90, and 100% 1RM) DV: Explosive strength of lower limbs	SJ: h (cm)	60% 1RM: 1 × 1 × 60% 1RM, rest 3 min + SJ. 70% 1RM: 1 × 1 × 70% 1RM, rest 3 min + SJ. 80% 1RM: 1 × 1 × 80% 1RM, rest 3 min + SJ. 90% 1RM: 1 × 1 × 90% 1RM, rest 3 min + SJ. 100% 1RM: 1 × 1 × 100% 1RM, rest 3 min + SJ.	60% 1RM: SJ (cm): pre = 54.37 ± 6.0 vs. 60% 1RM = 56.14 ± 5.0, Cohen’s *d* = 0.32 70% 1RM: SJ (cm): pre = 54.37 ± 6.0 vs. 70% 1RM = 56.39 ± 6.0, Cohen’s *d* = 0.34 80% 1RM: SJ (cm): pre = 54.37 ± 6.0 vs. 80% 1RM = 57.13 ± 5.0, Cohen’s *d* = 0.50 90% 1RM: SJ (cm): pre = 54.37 ± 6.0 vs. 90% 1RM = 55.99 ± 6.0, Cohen’s *d* = 0.27 100% 1RM: SJ (cm): pre = 54.37 ± 6.0 vs. 100% 1RM = 55.96 ± 5.0, Cohen’s *d* = 0.29	60% 1RM = ↑ 70% 1RM = ↑ 80% 1RM = ↑ 90% 1RM = ↑ 100% 1RM = ↑
Hirayama K [33]	To examine the acute effects of an ascending intensity squat protocol consisting of single-repetition exercises on subsequent vertical jump performance.	College weightlifters: M = 14 (19.9 ± 1.4 years) EG = 14 CG = 14 The participants completed two conditions on separate days in random order	IV: I-SVR DV: Explosive strength of lower limbs	CMJ: h (cm)	EG (isometric contractions + CMJ): CMJ BL + warm-up + CMJ post-warm-up + stretch + CMJ post-stretch + rest 3 min + 1 × 1 × 20% 1RM + CMJ + rest 3 min + 1 × 1 × 40% 1RM + CMJ + rest 3 min + 1 × 1 × 60% 1RM + CMJ + rest 3 min + 1 × 1 × 80% 1RM + CMJ + rest 3 min + 1 × 1 × 100% 1RM isometric + CMJ. CG (CMJ): CMJ BL + warm-up + CMJ post-warm-up + stretch + CMJ’ post-stretch + rest 3 min + CMJ + rest 3 min + CMJ + rest 3 min + CMJ + rest 3 min + CMJ + rest 3 min + CMJ.	EG1 vs. CG CMJ (m): ANOVA: *p* ˂ 0.001 Post hoc: EG: ANOVA *p* ˂ 0.001 CMJ post-stretch vs. 60% 1RM: *p* = 0.004 CMJ post-stretch vs. 80% 1RM: *p* ˂ 0.001 CMJ post-stretch vs. 100% 1RM isometric: *p* ˂ 0.001	EG ↑ CG: ↔
Li et al. [34]	To compare the effect of CT vs. HRT on strength and power indicators, running economy, and 5-km performance.	Well-trained male distance runners: EG1 = 10 (20.2 ± 1.0 years) EG2 = 9 (21.2 ± 1.4 years) CG = 9 (20.7 ± 1.2 years)	IV: I-SVR CT and HRT DV: Explosive strength of lower limbs	CMJ: h (cm)	EG1 CT: 8-week training intervention: CT + endurance training. EG2 heavy resistance training: 8-week training intervention: HRT + endurance training. CG: Strength-endurance training + endurance training.	EG1: CMJ (cm): pre = 31.06 ± 3.4 vs. post = 34.51 ± 3.8, *p* ˂ 0.001 EG2: CMJ (cm): pre = 32.80 ± 4.3 vs. post = 35.58 ± 3.3, *p* ˂ 0.001 CG: CMJ (cm): pre = 33.46 ± 4.7 vs. post = 34.26 ± 4.2, *p* > 0.05	EG1: ↑ EG2: ↑ CG: ↔
Okuno et al. [35]	To analyze the changes in RSA performance after heavy load exercise (crossover).	Elite handball players: M = 12 (18.7 ± 1.7 years) EG = 12 BL = 12 The participants completed two conditions on separate days in random order	IV: I-SVR CT DV: Explosive strength of lower limbs	T: RSA (s)	EG CT—back squat: 1 × 5 × 50% 1MR + 1 × 3 × 70% 1MR + 5 × 1 90% 1MR + RSA test BL: RSA test.	RSA mean (s): EG = 5.99 ± 0.19 vs. CG = 6.06 ± 0.18, *p* ˂ 0.01	EG: ↑
Suchomel et al. [36]	To compare the temporal profile of strong and weak subjects during ballistic and non-ballistic potentiation complexes (randomized).	Resistance-trained: M = 16 SG = 8 (23.5 ± 1.9 years) WG = 8 (25.1 ± 5.7 years)	IV: I-SVR CT DV: Explosive strength of lower limbs	SJ: h (cm)	P1—ballistic potentiation: 1 × 5 × 30% 1RM, rest 3 min, + 1 × 3 × 50% 1RM, rest 4 min, + 1 × 3 × 70% 1RM, rest 4 min, + 1 × 2 × 90% 1RM + 10 SJ (1 every minute). P2—non-ballistic potentiation: 1 × 5 × 30% 1RM, rest 3 min, + 1 × 3 × 50% 1RM, rest 4 min, + 1 × 3 × 70% 1RM, rest 4 min, + 1 × 2 × 90% 1RM + 10 SJ (1 every minute).	P1—ballistic potentiation: *p* = 0.44 P2—non-ballistic potentiation: *p* = 0.13	SG1: ↔ WG1: ↔
Intra-Set Variable Resistance							
Chiu & Salem [37]	To determine the acute effects of weightlifting on vertical jump joint kinetics, performance was assessed before, during, and after snatch pull exercises in male athletes.	Well-trained athletes: M = 13 (27.3 ± 4.2 years)	IV: I-sVR DV: Explosive strength of lower limbs	CMJ: h (cm)	EG (snatch pull): 2 × 4 (1 × 2 × 70% 1RM, rest 3 min + 1 × 2 × 80% 1RM, rest 3 min + 1 × 2 × 90% 1RM, rest 3 min + 1 × 2 × 100% 1RM). CMJ before, during, and after snatch-pull protocol.	EG–CMJ (cm): Before, during, and after snatch pull protocol (ANOVA): *p* ˂ 0.001. Post hoc: Pre-protocol vs. middle protocol: *p* ˂ 0.001, ES = 1.62. Pre-protocol vs. post-protocol: *p* ˂ 0.001, ES = 1.75. Middle protocol vs. post-protocol: *p* = 0.94, ES = 0.13.	EG: ↑
Crum et al. [38]	To examine the effects of a moderately loaded (50–65% of 1RM) concentric-only quarter back squat protocol on the occurrence of potentiation effects at various time points.	Well-trained athletes: M = 20 (22.1 ± 4.0 years) EG1 = 20 EG2 = 20 CG = 20 The participants completed three conditions on separate days in random order	IV: I-sVR DV: Explosive strength of lower limbs	CMJ: peak power (W)	EG1 (1/4 squats 50% 1RM): Warm-up + rest 2 min + CMJ, rest 1 min + 1 × 1 × 30% 1RM + 1 × 1 × 40% 1RM + 1 × 1 × 50% 1RM + CMJ (0.5, 3-, 5-, 10-, and 15-min post-treatment). EG2 (1/4 squats 65% 1RM): Warm-up + rest 2 min + CMJ, rest 1 min + 1 × 1 × 30% 1RM + 1 × 1 × 40% 1RM + 1 × 1 × 65% 1RM + CMJ (0.5-, 3-, 5-, 10-, and 15-min post-treatment). CG: Warm-up + rest 2 min + CMJ, rest 7 min + CMJ (0.5, 3, 5, 10, and 15 min).	EG1 vs. EG2 vs. CG peak power (W): ANOVA: *p* = 0.56, ES = 0.07 Post hoc: EG1: Pretrial vs. 30 s, *p* > 0.05; pretrial vs. 3 min *p* ˂ 0.01; pretrial vs. 5 min *p* ˂ 0.01; pretrial vs. 10 min *p* ˂ 0.01; pretrial vs. 15 min *p* ˂ 0.01. EG2: Pretrial vs. 30 s, *p* > 0.05; pretrial vs. 3 min *p* > 0.05; pretrial vs. 5 min *p* > 0.05; pretrial vs. 10 min *p* > 0.051; pretrial vs. 15 min *p* ˂ 0.01. CG: Pretrial vs. 30 s, *p* > 0.05; pretrial vs. 3 min *p* ˂ 0.05; pretrial vs. 5 min *p* ˂ 0.05; pretrial vs. 10 min *p* ˂ 0.051; pretrial vs. 15 min *p* ˂ 0.01.	EG1 vs. EG2 vs. CG: ↔ EG1: ↑ EG2: ↔ CG: ↑
Naclerio et al. [39]	To examine the acute effects of different parallel squat post-activation potentiation protocols with and without whole-body vibration on jumping performance in college athletes.	College athletes M = 15 (20.3 ± 1.3 years) EG1 = 15 EG2 = 15 CG = 15 The participants completed three conditions on separate days in random order	IV: I-sVR DV: Explosive strength of lower limbs	CMJ: h (m)	EG1 (parallel squat 80% 1RM without vibration): CMJ + 1 × 3 (low volume) + rest 1 min + CMJ + rest 3 min + CMJ and CMJ + 3 × 3 (high volume) + rest 1 min + CMJ + rest 3 min + CMJ EG2 (parallel squat 80% 1RM on a whole-body vibration platform): CMJ + 1 × 3 (low volume) + rest 1 min + CMJ + rest 3 min + CMJ and CMJ + 3 × 3 (high volume) + rest 1 min + CMJ + rest 3 min + CMJ CG: CMJ + rest 1 min + CMJ + rest 3 min + CMJ and CMJ + rest 1 min + CMJ + rest 3 min + CMJ	EG1 vs. EG2 vs. CG CMJ (m): ANOVA: *p* = 0.005, ES = 0.60 Post hoc: EG1: Low volume, *p* = 0.015; high volume, *p* > 0.05	EG1 vs. EG2 vs. CG: ↑ EG1: ↑ EG2: ↔ CG: ↔
Ojeda et al. (1) [40]	To determine the acute effect of CT on the bench press (intra-subject) on grenade-throwing velocity.	Military pentathletes: M = 19 (24.8 ± 5.3 years) EG = 19 BL = 19 Participants were compared to their BL	IV: I-sVR CT DV: Explosive strength of upper limbs	V: throw (km·h^−1^)	EG CT—bench press: 4 × (5 × 30% 1RM + 4 × 60% 1RM + 3 grenade throws, rest 15 s between throw), rest 3 min. BL: Three grenade throws (rest 15 s between throws).	EG1 (p = 0.94): Pair a—V (km·h^−1^): BL = 60.1 ± 7.2 vs. S1 = 60.4 ± 6.2, *p* = 0.52 Pair b—V (km·h^−1^): BL = 60.1 ± 7.2 vs. S2 = 60.2 ± 7.0, *p* = 0.45 Pair c—V (km·h^−1^): BL = 60.1 ± 7.2 vs. S3 = 59.7 ± 7.6, *p* = 0.32 Pair d—V (km·h^−1^): BL = 60.1 ± 7.2 vs. S4 = 59.0 ± 8.0, *p* = 0.13	EG: ↔
Ojeda et al. (2) [41]	To determine the acute effect of a CT in bench press on grenade throwing.	Professional and amateur military pentathletes (M = 19): PG = 10 (28.5 ± 4.8 years) AG = 9 (20.8 ± 1.6 years)	IV: I-sVR CT DV: Explosive strength of upper limbs	D: throw (m)	PG CT—bench press: 4 × (5 × 30% 1RM + 4 × 60% 1RM + 3 grenade throws, rest 15 s between throw), rest 3 min. AG CT—bench press: 4 × (5 × 30% 1RM + 4 × 60% 1RM + 3 grenade throws, rest 15 s between throw), rest 3 min.	PG (*p* = 0.001): Pair a—D (m): BL = 40.6 ± 6.0 vs. S1 = 39.0 ± 4.8, *p* = 0.07 Pair b—D (m): BL = 40.6 ± 6.0 vs. S2 = 41.5 ± 5.5, *p* = 0.01 Pair c—D (m): BL = 40.6 ± 6.0 vs. S3 = 41.6 ± 5.6, *p* = 0.26 Pair d—D (m): BL = 40.6 ± 6.0 vs. S4 = 42.1 ± 5.9, *p* = 0.01 AG (*p* = 0.012): Pair a—D (m): BL = 36.1 ± 6.8 vs. S1 = 33.9 ± 7.3, *p* = 0.01 Pair b—D (m): BL = 36.1 ± 6.8 vs. S2 = 34.1 ± 6.1, *p* = 0.29 Pair c—D (m): BL = 36.1 ± 6.8 vs. S3 = 33.6 ± 6.6, *p* = 0.25 Pair d—D (m): BL = 36.1 ± 6.8 vs. S4 = 31.3 ± 7.1, *p* = 0.75	PG: ↑ AG: ↓
Ojeda et al. (3) [42]	To determine the acute effect temporal of a CT protocol (intra-subject) on 30-m sprint times.	Military athletes: M = 7 (25.0 ± 2.6 years) EG = 7 BL = 7 Participants were compared to their BL	IV: I-sVR CT DV: Explosive strength of lower limbs	T: 30-m sprint (s)	EG CT—back squat: 4 × (5 × 30% 1RM + 4 × 60% 1RM + 3 sprint 30 m, rest 120 s between sprint), rest 3 min. BL: Three sprints 30 m (rest 120 s between sprints).	EG1 (*p* ˂ 0.0001): Pair a—T (s): BL = 4.57 ± 0.23 vs. S1 = 4.22 ± 0.20, *p* ˂ 0.01 Pair b—T (s): BL = 4.57 ± 0.23 vs. S2 = 4.27 ± 0.20, *p* ˂ 0.01 Pair c—T (s): BL = 4.57 ± 0.23 vs. S3 = 4.23 ± 0.23, *p* ˂ 0.01 Pair d—T (s): BL = 4.57 ± 0.23 vs. S4 = 4.23 ± 0.21, *p* ˂ 0.01	EG: ↑
Ojeda et al. (4) [43]	To determine the variations in the blood muscular damage indicators post application of two CT (intra-subject) for back squats.	Military athletes: M = 7 (25.0 ± 2.6 years) EG1 = 7 EG2 = 7 BL = 7 The participants completed two conditions on separate days in random order and were compared to their BL	IV: I-sVR CT DV: Explosive strength of lower limbs Muscular damage indicators	T: 30-m sprint (s) CK	EG1 CT—back squat: P1: 4 × (5 × 30% 1RM + 4 × 60% 1RM + 3 sprint 30 m, rest 120 s between sprint), rest 3 min. EG2 CT—back squat: P2: 4 × (4 × 60% 1RM + 5 × 30% 1RM + 3 sprint 30 m, rest 120 s between sprint), rest 3 min. BL: Three sprints 30 m (rest 120 s between sprints).	EG1: T (s): BL = 4.57 ± 0.23 vs. S1 = 4.22 ± 0.20, S2 = 4.27 ± 0.20, S3 = 4.23 ± 0.23, and S4 = 4.23 ± 0.21, *p* ˂ 0.001 CK-MB (U/L): BL = 20.7 ± 3.7 vs. P1 = 23.2 ± 6.4, *p* ˂ 0.05 CK-Total (U/L): BL = 145.7 ± 37.5 vs. P1 = 312.0 ± 137.2, *p* ˂ 0.05 EG2: T (s): BL = 4.57 ± 0.23 vs. S1 = 4.26 ± 0.17, S2 = 4.28 ± 0.17, S3 = 4.22 ± 0.16, and S4 = 4.22 ± 0.10, *p* ˂ 0.001 CK-MB (U/L): BL = 20.7 ± 3.7 vs. P1 = 24.1 ± 4.4, *p* ˂ 0.01 CK-Total (U/L): BL = 145.7 ± 37.5 vs. P1 = 301.1 ± 96.3, *p* ˂ 0.01	EG1: ↑ EG2: ↑
Ojeda et al. [44]	To determine the behavior of the following blood serum substances in a CT session: MB-CK and CK-Total.	Military athletes: M = 10 (28.5 ± 4.8 years) EG = 10 BL = 10 Participants were compared to their BL	IV: I-sVR CT DV: Muscular damage indicators Explosive strength of upper limbs	CK D: throw (m)	EG CT—bench press: 4 × (5 × 30% 1RM + 4 × 60% 1RM + 3 grenade throws, rest 15 s between throw), rest 3 min. BL: Three grenade throws (rest 15 s between throws).	EG: CK-MB (U/L): BL (pre) = 22.8 ± 7.9 vs. post = 20.0 ± 2.8, *p* = 0.23 CK-Total (U/L): BL (pre) = 233.4 ± 178.4 vs. post = 209,6 ± 74.2, *p* ˂ 0.64 D (m): BL = 36.1 ± 6.8 vs. S1 = 33.9 ± 7.3, S2 = 34.1 ± 6.1, S3 = 33.6 ± 6.6, and S4 = 31.3 ± 7.1, *p* ˂ 0.05	EG: ↓
Ojeda et al. (1) [45]	To determine the acute effect of an I-SVR protocol in back squats over time in 30-m sprints in sprinter women.	Sprinter women: W = 10 (20.3 ± 1.9 years) EG = 10 BL = 10 Participants were compared to their BL	IV: I-sVR CT DV: Explosive strength of lower limbs	T: 30-m sprint (s)	EG CT—back squat: 4 × (5 × 22% 1RM + 4 × 60% 1RM + 3 sprint 30 m, rest 120 s between sprint), rest 3 min. BL: Three sprints 30 m (rest 120 s between sprints).	EG: D (m): BL = 4.60 ± 0.23 vs. S1 = 4.58 ± 0.23, S2 = 4.61 ± 0.22, S3 = 4.60 ± 0.23, S4 = 4.59 ± 0.19, *p* > 0.05	EG: ↔
Ojeda et al. (2) [46]	To determine the behavior of CK before and after the execution of a pre-activation protocol with I-sVR to generate PAP.	Sprinter women: W = 6 (20.4 ± 2.0 years) EG1 = 6 EG2 = 6 BL = 6 The participants completed two conditions on separate days in random order and were compared to their BL	IV: I-sVR CT DV: Explosive strength of lower limbs Muscular damage indicators	T: 30-m sprint (s) CK	EG1 CT—back squat (with I-sVR): P1: 3 × (5 × 30% 1RM + 4 × 60% 1RM + 3 sprint 30 m, rest 120 s between sprint), rest 3 min. EG2 (without I-sVR): P2: 4 × 3 sprint 30 m (rest 120 s between sprint), rest 3 min. BL: Three sprints 30 m (rest 120 s between sprints).	EG1: T (s): BL = 4.73 ± 0.22 vs. S1 = 4.45 ± 0.17, S2 = 4.49 ± 0.19, and S3 = 4.45 ± 0.10, *p* ˂ 0.05 CK-MB (U/L): BL (pre)= 17.2 ± 3.3 vs. post = 24.7 ± 8.3, *p* ˂ 0.05 CK-Total (U/L): BL (pre) = 151.0 ± 39.3 vs. post = 575.5 ± 384.0, *p* ˂ 0.05 EG2: T (s): BL = 4.73 ± 0.22 vs. S1 = 4.58 ± 0.20, S2 = 4.52 ± 0.24, and S3 = 4.75 ± 0.43, *p* > 0.05 CK-MB (U/L): BL (pre)= 17.2 ± 3.3 vs. post = 22.5 ± 3.0, *p* ˂ 0.05 CK-Total (U/L): BL (pre) = 151.0 ± 39.3 vs. post = 572.8 ± 254.7, *p* ˂ 0.05	EG: ↑ CG: ↔
Intra-Repetition Variable Resistance							
Martínez-García et al. [47]	To investigate the acute effect of pre-activation with I-RVR and isometry on the overhead throwing velocity in handball players.	Handball players: F = 14 (21.2 ± 2.7 years) EG1: 14 EG2: 14 BL: 14 The participants completed two conditions on separate days in random order and were compared with their BL	IV: I-RVR DV: Explosive strength of upper limbs	V: throw (km·h^−1^)	EG1 (I-RVR unilateral chest press): Throw (BL) + 1 × 5 at an initial velocity of 0.6 m·s^−1^ and a final velocity of 0.9 m·s^−1^ + throw (0-, 1-, 2-, and 10-min post activation—post-test). EG2 (isometric unilateral chest press): Throw (BL) + 5 s voluntary maximum isometric contraction + throw (0-, 1-, 2-, and 10-min post activation—post-test). BL: Three grenades (rest 15 s between throws).	EG1 vs. EG2 throw (km·h^−1^): ANOVA: *p* > 0.05, ES = 0.08	EG1: ↔ EG2: ↔
Scott et al. [48]	To examine the PAP response of 2 conditioning activities, the hex bar deadlift and back squat, combined with accommodating resistance.	Amateur rugby players: M = 20 (22.3 ± 2.6 years) EG1 = 12 EG2 = 12 BL = 12 The participants completed two conditions on separate days in random order and were compared with their BL	IV: I-RVR DV: Explosive strength of lower limbs	CMJ: h (cm)	EG1—hex bar deadlift: 1 × 3 × 70% 1RM + elastic band (0–23% 1RM), rest 30 s, CMJ, rest 90 s, CMJ, and rest 180 s, CMJ. EG2—back squat: 1 × 3 × 70% 1 RM + elastic band (0–23% 1RM), rest 30 s, CMJ, rest 90 s, CMJ, and rest 180 s, CMJ. BL: Three CMJ.	EG1 (*p* > 0.05): Pair a—CMJ (m): BL vs. 30 s, *p* = 0.003 Pair b—CMJ (m): BL vs. 90 s, *p* > 0.05 Pair c—CMJ (m): BL vs. 180 s, *p* > 0.05 EG2 (*p* > 0.05): Pair a—CMJ (m): BL vs. 30 s, *p* = 0.005 Pair b—CMJ (m): BL vs. 90 s, *p* > 0.05 Pair c—CMJ (m): BL vs. 180 s, *p* > 0.05	EG1: ↑ (30 s) EG1: ↔ (90 s) EG1: ↔ (180 s) EG2: ↑ (30 s) EG2: ↔ (90 s) EG2: ↔ (180 s)
Wyland et al. [49]	To determine whether short sprints can be acutely enhanced after several sets of back squats with or without accommodating resistance.	Recreationally resistance-trained: M = 20 (23.3 ± 4.4 years) EG1 = 20 EG2 = 20 CG = 20 The participants completed three conditions on separate days in random order	IV: I-RVR DV: Explosive strength of lower limbs	T: 9.1-m sprint (s)	EG1 without elastic band: Warm-up + 3 × 9.1 m sprint (pre-test) + 5 × 3 back squat 85% 1RM + 5 × 9.1, sprint (0, 1-, 2-, 3-, and 4 min post-activation—post-test) EG2 with an elastic band: Warm-up + 3 × 9.1 m sprint (pre-test) + 5 × 3 back squat 85% 1RM (with 30% of the total load coming from accommodating resistance) + 5 × 9.1, sprint (0, 1-, 2-, 3-, and 4 min post-activation—post-test) CG: Warm-up + 3 × 9.1 m sprint (pre-test) + rest 10 min + 5 × 9.1, sprint (0, 1-, 2-, 3-, and 4-min post-activation—post-test)	EG1 vs. EG2 vs. CG 9.1 m sprint (s): ANOVA: *p* > 0.05 Post hoc: EG1: All comparisons *p* > 0.05 EG2: 9.1 m sprint to 0 min vs. 4 min post-activation *p* = 0.002 CG: All comparisons *p* > 0.05	EG1: ↔ EG2: ↑ CG: ↔

AG, amateur group; BL, baseline; CG, control group; CK, creatine kinase; CK-MB, metabolic creatine kinase; cm, centimeters, CMJ, counter movement jump; CT, complex training; D, distance; Cohen’s d, effect size; DJ, drop jump; DV, dependent variable; EG, experimental group; EG1, experimental group1; EG2, experimental group 2; ES, effect size; h, height; HRT, heavy resistance training; IV, independent variable; I-SVR, intra-session variable resistance; I-sRV, intra-set variable resistance; I-RVR, intra-repetition variable resistance; km·h−1, kilometers per hour; M, men; m, meters; min, minutes; *p*, *p*-value; PAP, post activation potentiation; PG, professional group; P1, protocol 1; P2, protocol 2; RDF, rate of force development; RPD, rate of power development; RSA, repeated-sprint ability; s, seconds; S, set; SG, strong group; SJ, squat jump; T, time; U/L, units per liter; V, velocity; vs., versus; W, women; WS, weak group; 1RM, one repetition maximum; ↑, performance increase; ↓, performance decline; ↔, performance maintenance.

## Data Availability

To see the results by keyword in each database, go to the following link: https://figshare.com/articles/dataset/Sources_of_information_and_research/22140602 (accessed on 23 February 2023).
